# Supplementary material for: Vernalization Procedure of Tuberous Roots Affects Growth, Photosynthesis and Metabolic Profile of Ranunculus asiaticus L
Source: Plants (Basel). 2023 Jan 17;12(3):425. doi: 10.3390/plants12030425 (PMC9920070; doi:10.3390/plants12030425)
Supplement: Supplementary file 1 [file plants-12-00425-s001.zip › plants-2028694-supplementary.pdf]

Supplementary Table (S1) Minor amino acids (in µmol g^{-1} DW) in plants of *Ranunculus asiaticus* L. hybrids MDR and MBO, obtained by three vernalization procedures of tuberous roots, only rehydration (Control, C), rehydration plus vernalization for 2 weeks (V2), rehydration plus vernalization for 4 weeks (V4). Week 8 from planting (vegetative phase). ns, *, ** and ***; indicate non-significant or significant difference at $p \leq 0.05$, $p \leq 0.01$, $p \leq 0.001$, respectively. Different lowercase or capital letters within each row, for specific vernalization procedure, indicate significant differences ($p \leq 0.05$).

Vegetative phase	**MDR**				**MBO**				**H**	**V**	**H x V**
	**C**	**V2**	**V4**	***Mean***	**C**	**V2**	**V4**	***Mean***			
Arg	0.56 a	0.94 b	1.01 b	*0.84*	0.69 a	1.13 b	0.63 a	*0.81*	ns	**	***
Hys	3.33	4.46	5.28	*4.36*	4.46	5.26	3.53	*4.42*	ns	ns	ns
Ile	0.98 a	0.78 ac	0.76 bc	*0.84*	0.95 a	0.90 ab	0.68 bc	*0.84*	ns	**	*
Leu	0.76 a	0.84 a	0.80 a	*0.80*	0.93 a	1.69 c	0.40 d	*1.01*	ns	ns	***
Lys	0.53 a	0.47 a	0.40 ac	*0.47*	0.37 ac	1.16 b	0.24 c	*0.59*	ns	*	***
Met	0.24 a	0.26 a	0.46 b	*0.32*	0.26 a	0.71 c	0.05 d	*0.34*	ns	ns	***
Phe	0.60 a	0.45 b	0.38 b	*0.48*	0.68 a	1.17 c	0.28 d	*0.71*	ns	**	***
Tyr	1.10 ab	0.96 bcd	1.35 a	*1.14 A*	0.62 c	0.70 dc	1.04 abd	*0.79 B*	**	ns	**
Trp	0.26 a	0.23 a	0.58 b	*0.36*	0.30 a	0.38 a	0.24 a	*0.31*	ns	ns	**
Val	1.39	1.40	1.58	*1.46*	1.32	1.61	1.21	*1.38*	ns	ns	ns
Minor AA	9.76 a	10.80 ac	12.60 ab	*11.05*	10.57 ac	14.71 c	8.30 c	*11.19*	ns	ns	**
Flowering phase	**MDR**				**MBO**				**H**	**V**	**H x V**
	**C**	**V2**	**V4**	***Mean***	**C**	**V2**	**V4**	***Mean***			
Arg	0.55 a	0.54 a	0.34 b	*0.48*	0.29 c	0.41 a	0.65 a	*0.45*	ns	ns	*
Hys	4.85 a	2.89 b	3.52 bc	*3.75*	3.12 b	3.02 b	4.07 c	*3.40*	ns	ns	***
Ile	0.46 a	0.53 a	0.29 b	*0.43*	0.24 c	0.37 a	1.31 d	*0.64*	ns	ns	**
Leu	0.27 a	0.32 a	0.17 b	*0.25 A*	0.40 c	0.51 d	1.17 e	*0.69 B*	***	ns	**
Lys	0.42 ac	0.27 bd	0.17 bd	*0.29*	0.21 d	0.36 ab	0.53 c	*0.37*	ns	ns	***
Met	0.05 a	0.07 ab	0.04 a	*0.05 A*	0.09 bc	0.14 d	0.11 cd	*0.11 B*	***	ns	***
Phe	0.49 a	0.24 a	0.14 b	*0.29*	0.23 a	0.27 a	1.19 c	*0.56*	ns	ns	*
Tyr	0.57 ab	0.67 ab	0.25 b	*0.50*	0.42 ab	0.45 ab	1.47 c	*0.78*	ns	ns	***
Trp	0.35 a	0.38 a	0.09 b	*0.27*	0.54 a	0.17 c	0.31 a	*0.34*	ns	*	*
Val	0.92 a	0.79 ac	0.38 b	*0.70*	0.43 b	0.66 c	1.84 d	*0.98*	ns	ns	***
Minor AA	8.94 a	6.69 b	5.40 b	*7.01*	5.97 b	6.36 b	12.64 c	*8.32*	ns	ns	***
